# Exploring facilitators of the implementation of electronic health records in Saudi Arabia

**DOI:** 10.1186/s12911-022-02072-5

**Published:** 2022-12-07

**Authors:** Haitham A. Alzghaibi, Hayley A. Hutchings

**Affiliations:** 1 Department of Health Informatics, College of Public Health and Health Informatics, Qassim University, 52741 Albukayriah, Saudi Arabia; 2 Medical School, Swansea University, Singleton Park, Swansea, SA2 8PP UK

**Keywords:** Electronic health records system, Facilitators, Primary Health Care Centres, General practitioners

## Abstract

**Introduction:**

The introduction of information technology was one of the key priorities for policy-makers in health care organisations over the last two decades due to the potential benefits of this technology to improve health care services and quality. However, approximately 50% of those projects failed to achieve their intended aims. This was a result of several factors, including the cost of these projects. The Saudi Ministry of Health (MoH) planned to implement an electronic health record system (EHRS) in approximately 2100 primary health care centres nationwide. It was acknowledged that this project may face hurdles, which might result in the failure of the project if implementation facilitators were not first determined. According to the Saudi MoH, previous electronic health record system implementation in primary health care centres failed as a consequence of several barriers, such as poor infrastructure, lack of connectivity and lack of interoperability. However, the facilitators of successful electronic health record system implementation in Saudi primary health care centres are not understood.

**Aim:**

To determine the facilitators that enhance the success of the implementation of an EHRS in public primary health care centres in SA.

**Method:**

A mixed methods approach was used with both qualitative and quantitative methods (qualitative using semistructured interviews and quantitative with a closed survey). The purpose of the utilisation of exploratory mixed methods was to identify a wide range of facilitators that may influence EHRS implementation. The data were obtained from two different perspectives, primary health care centre practitioners and project team members. A total of 351 practitioners from 21 primary health care centres participated in the online survey, and 14 key informants at the Saudi Ministry of Health who were directly involved in the electronic health record system implementation in the primary health care centres agreed to be interviewed face to face.

**Results:**

The findings from both studies revealed several facilitators. Among these facilitators, financial resources were found to be the most influential factor that assisted in overcoming some barriers, such as software selection. The size of the primary health care centres was the second facilitator of successful implementation, despite the scale of the project. Perceived usefulness was another facilitator identified in both the interviews and the survey. More than 90% of the participants thought that the electronic health record system was useful and could contribute to improving the quality of health care services. While a high level of satisfaction was expressed towards the electronic health record system’s usability and efficiency, low levels of satisfaction were recorded for organisational factors such as user involvement, training and support. Hence, system usability and efficiency were documented to be other facilitators of successful electronic health record system implementation in Saudi primary health care centres.

**Conclusion:**

The findings of the present study suggest that sufficient financial support is essential to enhance the success of electronic health record system implementation despite the scale of the project. Additionally, effective leadership and project management are core factors to overcome many obstacles and ensure the success of large-scale projects.

## Introduction

Since the 1960s, information technology (IT) has been responsible for the performance enhancement and improvement of health care services [1–4]. The implementation of IT in the last few decades of the twentieth century led to a revolution in the way work is performed and the way information is categorised and documented. The speed and precision of the IT revolution made the governments of developed countries (where this revolution originated) immediately adopt these advanced, fast and efficient systems [4, 5]. As a result, the implementation of electronic health record systems (EHRSs) has become a priority for both developed and developing countries [5, 6]. An EHRS is “*a computerized health information system where providers record detailed encounter information such as patient demographics, encounter summaries, medical history, allergies, intolerances and lab test histories. Some may support order entry, results management and decision support*” [7].

Various researchers have argued that EHRS implementation is complicated due to the shortage of experience in implementation and associated issues [8–12]. Although barriers to EHRS implementation have been described, such as low-efficiency systems, lack of end-user involvement and privacy and confidentiality issues, many remain unresolved [13]. Therefore, further research and investigation are necessary to overcome these barriers [13]. According to Keshavjee, Bosomworth [14], Greenhalgh, Stramer [15], Lorenzi, Smith [16], and Pare, Sicotte [17], approximately 50% of EHRS implementation projects around the world have failed due to factors such as project leadership issues, usability issues and the cost of EHRS implementation projects. Others have estimated that the proportion of unsuccessful IT projects in the health care setting could be as high as 70%, which can be directly linked to factors such as user resistance and a lack of perceived usefulness [18]. According to Gagnon, Desmartis [19], the implementation of EHRS in primary health care centres (PHCs) remains a greater challenge than its implementation in secondary care, such as hospitals.

Across disciplines, at all levels, and throughout the world, it is recognised that the provision of health care is becoming increasingly complex [20], particularly in developing countries, due to infrastructure issues, organisational workflow issues and cost challenges [21]. Due to this complexity, the introduction of ICT in health care organisations poses many challenges [21]. According to Sanchez, Savin [20], one of the obstacles to EHRS implementation is the large number of health care practitioners working in these organisations, which complicates the process of EHRS implementation. Due to this complexity, several barriers have been recorded in Saudi Arabia (SA). According to El Mahalli [22], accessibility, training and support, and usability were the most cited barriers to EHRS implementation. Another study detected a low level of satisfaction among physicians and nurses with the use of EHRS in SA [23]. Overall, practically successful EHRS implementation is subject to several factors classified as the human-organisation-technology-fit (HOT-fit) framework [24, 25].

Knowledge about the usefulness and benefits of EHRS, as well as the implementation costs and other factors, is considered scant in developing countries [26]. Despite the fact that research on the impact of EHRS and its potential benefits has been conducted in developed countries, knowledge about the system’s impact is still conflicting [26]. Moreover, implementation in developing countries requires greater effort than in developed countries because the readiness of health care organisations is lower with respect to aspects such as IT and infrastructure [27, 28].

### Importance of EHRS

The positive impact of EHRS was noticed almost immediately in the field of primary health care [29]. It has been suggested that implementing EHRS will enhance the accessibility of and the process of distributing health records between authorised users [30–34]. In addition, EHRS can improve the economic and administrative abilities of all health care institutions and directly influence the quality of care provided to patients [35–39].

### Primary care centres

As part of a country’s medical system, PHCs, which are known globally as general practitioners (GPs), have attained paramount significance in the process of giving care. This fundamental care is critical to the proper delivery of health care [40]. Its importance lies in the fact that PHCs form the primary point of supervision and the foremost channel for the provision of medical care for a considerable percentage of the population [41]. To that end, PHCs are increasingly recognised as substantial alternatives to hospitals considering that the elderly population is growing phenomenally and that patient autonomy and independence are growing [42]. PHCs are a realistic channel for realising quality fundamental medical care that is comprehensive, publicly available and easily accessed by the general population [43]. In most countries, PHCs are where patients engage with their health system [44].

As part of its duties, the Saudi Ministry of Health (MoH) supervises more than 2100 PHCs divided among thirteen regions in SA. Each PHC provides health services for an estimated 13,455 people [45]. The automation of PHCs in SA is part of the Saudi e-health strategy [46]. According to [46], 2900 PHCs will be automated by the implementation of the EHRS. Three different vendors will play a role in the implementation of the EHRS among PHCs over ten years[46]. However, only two EHRSs from Saudi national vendors have been selected to date. These systems are known as “Wahed”, produced by the Elm company [47], and “Raqeem”, produced by the Lean company [48]. The selected systems are customised versions of commercial public health solutions that were originally developed by IQVIA.com [49]. As part of the national transformation programme, the Saudi MoH has adopted Health Level Seven (HL7) to achieve interoperable EHRSs in both primary and secondary care [50]. Therefore, both Wahed and Raqeem adhere to HL7.

### Aim of the study

To determine the facilitators that enhance the success of the implementation of the EHRS in public PHCs in SA.

## Methods

### Research ethics and governance

The participant information sheets for the questionnaire-based research explained the reasons for the study and why the participants had been approached. Participants were told that they could withdraw at any time even if they had initially agreed to participate. Furthermore, the participants were told that no benefits would be offered in return for their participation. The participants were informed that their participation in the study was valued and that it may help make the process of EHRS implementation within PHCs more efficient and effective. All participants were told that the data gathered would be fairly processed, analysed and published. The privacy and anonymity of the participants was assured. We indicated to the participants that they would encounter no mental or physical harm during the process of gathering data. In the final stage, our contact details were provided so that the participants could make contact if they had any concerns regarding the process and the subsequent treatment of the information gathered.

### Research design and data collection

A mixed-methods approach was used that included both qualitative semistructured interviews and a quantitative closed-ended survey. The purpose of the initial semistructured interviews was to explore and identify possible facilitators that may influence EHRS implementation and to use this information to design a more focused questionnaire for the quantitative survey study. We wanted to obtain different perspectives regarding EHRS implementation and, as such, recruited both PHC practitioners and project team staff. Examining the facilitators from the perspective of PHC practitioners was expected to provide information about factors that were related to personnel, such as end-user satisfaction. Factors related to the organisation, such as cost, training, and support, and factors related to technology, such as usability and efficiency, would be better obtained from the project team’s perspective. To achieve the aim of this study, we took into account the main factors influencing EHRS implementation as determined by previous literature during the creation of the questionnaire as well as findings obtained throughout the semistructured interviews.

Although previous literature was considered during the questionnaire design, to improve the questionnaire’s validity, the majority of questions were taken from a preexisting questionnaire, “*The Clinical Information System Implementation Evaluation Scale (CISIES)*” [51] The CISIES has been widely used in the field of ICT implementation in health care and has been implemented in various similar studies [e.g. 52]. CISIES can gather users’ attitudes on health information systems. Furthermore, the findings of the semistructured interviews improve the validity of the questionnaire. This assists in validating important findings from different perspectives and larger subjects. The implemented data collection approach was driven by sequential exploratory mixed methods [53].

### Study questionnaire validity and reliability

We conducted several preliminary procedures to assess and, if necessary, improve the questionnaire. The development of the questionnaire was conducted over two phases:

#### Phase one

The preliminary instrument was reviewed by two different expert panels. The first panel consisted of the supervisory team. The second panel consisted of external experts, including the following:

- two IT specialists from the headquarters of the Saudi MoH.
- the heads of the IT departments of two different hospitals.
- one academic from King Saudi University (who held a PhD in Health Informatics).
- one radiologist.
- one pharmacist (who held a master’s degree in Health Informatics).

The purpose of the expert panels was to review the preliminary instrument, to check whether the questions were understandable, and to assess the content of the questions to ensure that all included items were relevant to the target population and addressed the study objectives.

#### Phase two

Following a review and feedback on the questionnaire by the expert panels, it was sent to a small number of members (n = 5) of the project team as a pilot study to ensure that the questionnaire was clear, understandable, and reliable. The data collection questionnaire was piloted at the headquarters of the Saudi MoH with volunteer members from the project team who had agreed to take part. Certain criteria were taken into consideration, such as different position levels (e.g., supervisors, directors and senior managers), different departments, and different nationalities.

This study utilised two scales. This section measured the reliability of each of those scales (perceived usefulness of EHRS and positive attitude). As seen in Table 1, the reliability of all scales was high. The perceived usefulness scale showed the highest reliability (0.92), followed by positive attitudes (0.89).

**Table 1:** Scale reliability using Cronbach’s alpha

*Scale*	Number of Items	Cronbach’s Alpha
Perceived usefulness of the EHRS	17	0.92
Positive attitude	15	0.89
Entire questionnaire	32	0.83

The qualitative data were analysed using a thematic analysis approach with NVivo10 software produced by QSRInternational.com. The quantitative data were statistically analysed using IBM SPSS V.22. Initially, descriptive statistics were performed using the median, rank, and total agreement. Then, nonparametric tests were used to determine whether any differences existed between groups. The Mann‒Whitney U test was used for two groups, and the Kruskal‒Wallis test was used for three or more groups.

#### Population and sampling

To reach the most appropriate subjects for this study (taking into consideration their involvement in the project implementation and their knowledge about EHRS implementation in PHCs in SA), nonprobability purposive snowball sampling was used [53, 54]. For qualitative purposes, all project team members (n = 53) were invited to participate in the semistructured interviews; however, only 14 agreed to participate. The sampling strategy applied for quantitative study was a multistage cluster sampling technique [55, 56]. The Saudi MoH divides SA into thirteen regions [57]. Therefore, in Stage One, we utilised the same division adopted by the Saudi MoH, with regions converted to clusters (see Table 2) [57]. In the second stage, simple random sampling based on the geographical location of each province was used [53]. In Stage Three, a total of 21 out of 2259 PHCs were randomly selected within the five chosen clusters. The sample (n = 491) was drawn from the selected 21 PHCs across the five selected regions. The multistage cluster sampling technique has been found to be effective for statewide studies and larger populations. Moreover, multistage cluster sampling assists in overcoming random sampling challenges [53].

**Table 2:** Main regions in SA and the number of PHCs in each province

No.	Region	Geographical location	Number of PHCs	Selected in this study	Number of selected PHCs
1	Riyadh	East	435	Yes	6
2	Gassim	Centre	159	Yes	4
3	Makkah	West	355	Yes	5
4	Almadinah	West	154	No	
5	Alsharqiah	East	248	No	
6	Albaha	South	101	Yes	3
7	Asir	South	317	No	
8	Najran	South	65	No	
9	Hail	North	100	No	
10	Alshamaliyah	North	45	No	
11	Jazan	South	155	No	
12	Tabuk	North	73	No	
13	Aljouf	North	52	Yes	3
Total	13		2259		21

## Results

Fourteen project team members at the Saudi MoH who were directly involved in EHRS implementation in the PHCs agreed to be interviewed face to face, while 351 practitioners from 21 PHCs participated in the online-based survey. This equated to a response rate of 71.5%.

### Interview results

The participants’ occupations involved five different positions (see Table 3): General Manager (n = 3), Head of Department (n = 3), Deputy Head of Department (n = 1), Software Developer (n = 1), and Analyst (n = 5).

**Table 3:** Participant abbreviations

Position	Code used
General Manager	GM
Head of Department	HD
Deputy Head of Department	DHD
Software Developer	SD
Data Analyst	DA

The analysis of qualitative data identified six factors that assisted the project team during EHRS implementation in PHCs in SA. These facilitators included e-government, the characteristics of PHCs in SA, financial resource leadership and management, user willingness, and perceived usefulness of the EHRS.

### E-government trend in SA

There is a move by the Saudi MoH towards digital transformation. This trend is supported directly by the higher authority in SA, as highlighted in the Saudi Vision 2030:

“The main orientation of SA is transforming all government services into electronic transactions. This is an important factor which helped and encouraged the Ministry to implement EHRS in all sectors.” (GM 1)

### Financial resources (FRs)

The Saudi MoH is characterised by an abundance of financial resources provided by the Saudi government, and the participants agreed upon this unanimously. Overall, FRs have a very positive impact on EHRS implementation projects. All participants reported that FRs contributed positively and facilitated the success of many previous projects, particularly EHRS implementation projects, due to the country’s ability to fund electronic transformation in all sectors and services. For instance, the participants said,

“The role of FR is definitely positive; this country has more access to financial resources.” (DA 3)

“The financial resources are the most important factor that contribute to the success of the project.” (DA 1)

“The main factor which helps us to implement EHRS is financial support.” (SD 1)

### Characteristics of PHCs

PHCs in SA are very similar to each other in terms of the health care provided and business workflow. The business process, structure and workflow are considered to be the same in all PHCs in SA. This facilitates software selection and other processes of EHRS implementation.

“If you have a look at the PHCs, they all offer the same services and the same standards; there is no difference between them.” (HD 1)

“The PHCs in the Kingdom are similar, have the same characteristics and work in the same field.” (GM 2)

Moreover, PHCs in SA are distinguished by their small size and number of staff compared to other health care sectors in SA. The small size of Saudi PHCs allows greater flexibility to implement new projects and assist in overcoming challenges such as training.

“The small size and number of staff in PHCs is ideal for the training process. Services provided by the PHCs are easy when compared with hospital services, and that helps in the implementation of the system and training.” (HD3)

### Leadership and management

Management and leadership are instrumental in the success of an EHRS implementation project.

“Leadership and management have an important role and are essential to the success of any project. The most important thing that affects such projects is the support from leaders and managers.” (DA 1)

In this context, strong leadership has a very positive impact on EHRS implementation projects and contributes significantly to the success of any project, particularly when support comes from senior management and others who have authority and influence at the ministry level. Concurring with this view, one of the general managers claimed that the success of EHRS implementation projects is 50% dependent on strong support and leadership at the senior manager level. Due to its importance and great influence on the success or failure of EHRS implementation projects, leadership has been discussed at all conferences, meetings and workshops held at the Saudi MoH.

“Support from senior management is one of the most important facilitators of successful EHRS implementation.” (HD 1)

“EHRS implementation projects generally rely 50% on management and leadership.” (GM 1)

One of the benefits of strong leadership in EHRS implementation projects is that it ensures that there is no disruption or delay. In addition, it prevents complacency in relation to the completion of these projects and minimises any errors, as represented by the comment below:

“It certainly helps in the success of the system by providing adequate budgets, careful follow-ups and supervision and gives strong commands to ensure that there is no leniency or delay in the implementation of any project.” (HD 3)

Furthermore, the participants highlighted the positive role played by senior managers in driving the development wheel, especially in relation to participating in the implementation of an EHRS in PHCs. HD2 said, “No doubt, the senior management team is the foundation, and if they aren’t involved, the project may fail”. The highest authority in the Ministry, represented by the minister and his deputies, was involved in the EHRS implementation project.

“There was high-level participation of the Ministry in this project, and they have a big role in this project.” (DA 2)

### Perceived usefulness of an EHRS

The time and effort savings, cost and error reduction and disease control benefits of an EHRS were considered the most important factors that encourage senior managers to implement it.

“Senior management became aware of the role of IT and the extent of savings that could be achieved with the EHRS, whether in terms of money, time or effort. Senior management is also aware that the EHRS will help to reduce errors.” (GM 3)

### EHRS end-user willingness to use the system

End-user willingness plays an important role in EHRS implementation in Saudi PHCs.

“One of the main facilitators is the willingness of users themselves.” (DHD1)

### Questionnaire results

The questionnaire data were collected from 351 participants across five different regions of the Kingdom of SA. The largest number of respondents, 103 (29.3%), were residents of the capital city, Riyadh (see Table 4).

**Table 4:** Participation distribution based on geographical location

Region	Frequency	Percent
Riyadh	103	29.3
Gassim	61	17.4
Aljouf	69	19.7
Albaha	30	8.5
Makkah	88	25.1
Total	351	100.0

 All participants worked in health care and administrative roles. As seen in Tables 5 and 149 (42.4%) were in an administrative role, such as managers, secretaries and receptionists; 104 (29.6%) worked in a nursing role; thirty-two (9.1%) were physicians; and thirty (8.5%) were pharmacists. Four (1.1%) participants did not declare their occupation.

**Table 5:** Participant distribution based on occupation

Occupation	Frequency	Percent
Administrator	149	42.4
Physician	32	9.1
Nurse	104	29.6
Lab technician	11	3.1
Pharmacist	30	8.5
Radiologist	9	2.6
Dentist	12	3.4
Total	347	98.9

Age was measured via six categories, as illustrated in Table 6 below. The majority of participants, 192 (54.7%), were between twenty-five and thirty-four years of age. A detailed breakdown of the age categories is provided in Table 6. Four participants (1.1%) did not declare their age.

**Table 6:** Participant distribution based on age

Age Group	Frequency	Percent
18 to 24	3	0.9
25 to 34	192	54.7
35 to 44	123	35.0
45 to 54	23	6.6
55 to 64	4	1.1
65 to 74	2	0.6
Total	347	98.9

Participants were asked to specify their gender. Participants were mostly male (n = 261; 74.4%); out of 351 participants, only eighty-one (23.1%) were female. Nine participants (2.6%) did not declare their gender.

The participants’ experience of using a personal computer at home varied, with most participants, 129 (36.8%), stating that they had experience ranging between ten and fifteen years. Only eighteen participants (5.1%) had less than one year of experience using a personal computer (see Table 7). Four participants (1.1%) did not declare their experience using a personal computer at home.

**Table 7:** Participant distribution based on experience using a personal computer

Length of experience	Frequency	Percent
Less than 1 year	18	5.1
1 to 5 years	29	8.3
5 to 10 years	109	31.1
10–15 years	129	36.8
More than 20 years	62	17.7
Total	347	98.9

The participants’ time spent working in their current work role was measured via five categories. The majority of participants, 105 (29.9%), had one to five years of experience. A detailed breakdown of the participants’ time in their current position is provided in Table 8. Five participants (1.4%) did not declare their experience using a personal computer at home.

**Table 8:** Participant distribution based on experience in their current position

Length of experience	Frequency	Percent
Less than 1 year	19	5.4
1 to 5 years	105	29.9
5 to 10 years	100	28.5
10 to 15 years	82	23.4
More than 20 years	40	11.4
Total	346	98.6

### Perceived usefulness of the EHRS

The participants expressed a very high level of satisfaction with the usefulness of an EHRS. It was evident from the participants’ responses across all the items that they were satisfied with the usefulness of the implemented EHRS. Based on the questions in the second section of the questionnaire related to the benefits of using the EHRS, such as medication error reduction, cost reduction, improved patient safety and quality of care, there was a high level of agreement with all items, ranging from a high of 93.7% to a low of 87.3%. Table 9 shows that the items with the highest level of endorsement were (1) “EHRS reduces costs through decreased paperwork, improved safety, reduced duplication of testing and improved health” (93.6%); (2) “EHRS helps to promote legible documents” (93.1%); (3) “Sharing electronic information with patients and other clinicians is more secure when using the EHRS” (92.7%); and (4) “The EHRS helps to do streamlined coding” (92.7%). Items with a lower level of endorsement were 14) “Using the EHRS helps to provide safer care” (88.7%); 15) “Information from the EHRS enables me to make better decisions about patient care” (88.3%); 16) “Using the EHRS helps to effectively diagnose patients” (88.2%); and 17) “Using the EHRS helps to reduce medical errors” (87.3%).

**Table 9:** Degree of endorsement for each of the seventeen questions relating to the perceived usefulness of EHRS

Items		Strongly Disagree	Disagree	Neutral	Agree	Strongly Agree	Median	Total agreement	Rank
The EHRS reduces costs through decreased paperwork, improved safety, reduced duplication of testing and improved health care	N	5	4	4	87	105	5.00	192	1
	%	2.4	2.0	2.0	42.4	51.2		93.6	
The EHRS help to promote legible documents	N	4	6	4	94	95	4.00	189	2
	%	2.0	3.0	2.0	46.3	46.8		93.1	
Sharing electronic information with patients and other clinicians is more secure when using the EHRS	N	6	4	5	91	99	4.00	190	3
	%	2.9	2.0	2.4	44.4	48.3		92.7	
The EHRS helps with streamlined coding	N	4	6	5	97	93	4.00	190	4
	%	2.0	2.9	2.4	47.3	45.4		92.7	
The EHRS enables quick access to patient records for more coordinated and efficient care.	N	8	5	3	95	94	4.00	189	5
	%	3.9	2.4	1.5	46.3	45.9		92.2	
Using the EHRS improves patients’ and health care professionals’ interaction and communication as well as health care convenience	N	5	5	6	101	88	4.00	189	6
	%	2.4	2.4	2.9	49.3	42.9		92.2	
The EHRS allows me to spend more time on other aspects of patient care	N	6	6	5	94	94	4.00	188	7
	%	2.9	2.9	2.4	45.9	45.9		91.8	
The EHRS helps to provide accurate information	N	5	7	5	100	88	4.00	188	8
	%	2.4	3.4	2.4	48.8	42.9		91.7	
The EHRS enables safer and more reliable prescribing	N	4	6	8	88	99	4.00	187	9
	%	2.0	2.9	3.9	42.9	48.3		91.2	
The EHRS helps to have complete documentation	N	6	8	6	92	93	4.00	185	10
	%	2.9	3.9	2.9	44.9	45.4		90.3	
The EHRS provides accurate, up-to-date and complete information about patients at the point of care	N	5	8	7	106	79	4.00	185	11
	%	2.4	3.9	3.4	51.7	38.5		90.2	
The EHRS improves end-user productivity and efficiency	N	6	9	7	90	92	4.00	182	12
	%	2.9	4.4	3.4	44.1	45.1		89.2	
The EHRS improves the privacy and security of patient data	N	6	6	11	88	93	4.00	181	13
	%	2.9	2.9	5.4	43.1	45.6		88.7	
Using the EHRS helps to provide safer care	N	4	8	11	93	88	4.00	181	14
	%	2.0	3.9	5.4	45.6	43.1		88.7	
Information from the EHRS enables me to make better decisions about patient care	N	5	8	11	99	82	4.00	181	15
	%	2.4	3.9	5.4	48.3	40.0		88.3	
Using the EHRS helps to effectively diagnose patients	N	5	9	10	96	83	4.00	179	16
	%	2.5	4.4	4.9	47.3	40.9		88.2	
Using the EHRS helps to reduce medical errors	N	6	7	13	96	83	4.00	179	17
	%	2.9	3.4	6.3	46.8	40.5		87.3	

Positive attitudes towards the use of the EHRS.

Based on the responses to the fourteen items representing positive attitudes towards EHRS implementation and use, it was clear that there was a high level of positive endorsement. The highest level of endorsement was 97.5%, and the lowest was 79.6%. Looking at the items individually, those with the highest level of endorsement were (1) “Overall, I prefer using the EHRS to the paper-based system” (97.5%); (2) “The EHRS is more efficient than a paper-based system” (95.1%); and (3) “Using the EHRS leads to better adherence to policies and procedures” (92.6%). The items with the lowest level of endorsement (but still with more agreement than disagreement) were 13) “The EHRS takes into account the specific needs of my care area(s)” (81.9%); 14) “Overall, the introduction of the EHRS has been effective” (80%) and 15) “I’m committed to the successful use of the EHRS” (79.6%).

In terms of EHRS usability, efficiency and information quality, Table 10 below shows a high level of agreement towards all items representing EHRS usability: “The EHRS is easy to use” (86.2%) and “I am physically comfortable while using the EHRS equipment and hardware” (87.2%). Furthermore, a high level of agreement was recorded for items representing EHRS efficiency: “The EHRS is more efficient than a paper-based system” (95.1%). Table 10 also illustrates the level of agreement towards information quality items. High agreement was found for “I can depend on the accuracy of the EHRS” (93.6%) and “Information almost never gets lost in the EHRS” (83.3%).

**Table 10:** Responses and endorsement to fourteen statements on positive attitudes towards the use of the EHRS scale

Items		Strongly Disagree	Disagree	Neutral	Agree	Strongly Agree	Median	Total agreement	Rank
Overall, I prefer using the EHRS to the paper-based system	N	1	2	2	91	107	4	198	1
	%	0.5	1.0	1.0	44.8	52.7		97.5	
The EHRS is more efficient than a paper-based system	N		4	6	80	113	4	193	2
	%		2.0	3.0	39.4	55.7		95.1	
I can depend on the accuracy of the EHRS	N	1	6	6	104	85	4	189	3
	%	0.5	3.0	3.0	51.5	42.1		93.6	
Using the EHRS leads to better adherence to policies and procedures	N	3	5	7	108	81	4	189	4
	%	1.5	2.5	3.4	52.9	39.7		92.6	
The EHRS facilitates the communication of patient information among members of our health care team	N	2	4	13	87	99	4	186	5
	%	1.0	2.0	6.3	42.4	48.3		90.7	
I am physically comfortable while using the EHRS equipment and hardware	N	4	8	14	90	88	4	178	6
	%	2.0	3.9	6.9	44.1	43.1		87.2	
The EHRS has improved my practice	N	4	6	21	78	96	4	174	7
	%	2.0	2.9	10.2	38.0	46.8		84.8	
I feel the use of the EHRS has improved the quality of patient care	N	2	7	21	99	75	4	174	8
	%	1.0	3.4	10.3	48.5	36.8		85.3	
The EHRS is easy to use	N	3	7	18	104	70	4	174	9
	%	1.5	3.5	8.9	51.5	34.7		86.2	
I feel the use of the system has improved patient care outcomes	N	5	5	25	84	86	4	170	10
	%	2.4	2.4	12.2	41.0	42.0		83.0	
Information almost never gets lost in the EHRS	N	5	11	18	84	85	4	169	11
	%	2.5	5.4	8.9	41.4	41.9		83.3	
The EHRS takes into account the specific needs of my care area(s)	N	6	10	21	90	78	4	168	12
	%	2.9	4.9	10.2	43.9	38.0		81.9	
Overall, the introduction of the EHRS has been effective	N	8	9	24	79	85	4	164	13
	%	3.9	4.4	11.7	38.5	41.5		80.0	
I am committed to the successful use of the EHRS	N	12	7	23	86	77	4	163	14
	%	5.9	3.4	11.2	42.0	37.6		79.6	

## Discussion

The aim of this study was to determine the facilitators that enhance the successful implementation of the EHRS in public PHCs in SA. Consequently, this study was conducted to identify the potential facilitators to assist EHRS implementers in primary health care centres. Both semistructured and online surveys confirmed that financial resources have a very positive impact on facilitating implementation, especially on the provision of efficient systems, training and technical support, and infrastructure preparation. Moreover, our study recorded a positive correlation between the perceived usefulness of an EHRS and end-users’ willingness, which can facilitate project success. The most obvious finding that emerged from the analysis was the positive impact of the system’s usability. Our study reported other facilitators of EHRS implementation, including PHC strong leadership, the type of project management, and digital transformation trends.

### Financial resources (FRs)

The results illustrated that financial resources (FRs) had a very high positive impact on facilitating the implementation of large-scale EHRSs in the PHCs and contributing to overcoming many challenges. The findings showed that the Saudi MoH did not face any financial constraints during the implementation of the EHRS projects. Thus, the influence of this factor was examined against some of the main factors found to have a direct relationship with FRs. The factor most influenced by FRs was software selection; 93.5% of the project team agreed that FRs assist in the selection of high-quality software. It was perceived that FRs could have a beneficial effect on software selection, allowing more flexibility to select the best vendors to implement EHRSs in PHCs and enhance system interoperability. Although preparing adequate infrastructure is very costly [58], this factor was significantly influenced by the provision of FRs in a positive way. The provision of training and technical support has previously been reported as a barrier to implementing an EHRS [59]. 90% of the participants agreed that FRs have a very positive impact on the provision of training.

Although the findings of this study illustrated that FR was one of the main facilitators of the implementation of the EHRS in Saudi PHCs, other studies have found that the cost of implementation was one of the main barriers, and Saudi health care organisations struggle to support their projects due to FR shortages [60–62]. Internationally, the cost of EHRS implementation is classified as a barrier to the success of projects [e.g. 4, 37, 59, 63–70].

### Characteristics of PHCs

The PHC workflow and business structure, which is unified for all PHCs in SA, is another facilitator of EHRS implementation. This unification is due to the CM system adopted by the Saudi MoH. This unification facilitates software selection, where one system can be implemented in all PHCs in SA. In addition to software selection, training courses can be unified due to similarities in PHC health care functions, workflows and business structures.

Furthermore, the findings illustrate that the size of the PHCs in SA was a facilitator of EHRS implementation. Similarly, the size of the health care organisation has been recognised as an influential factor [71, 72]. In this regard, our results revealed that the project team benefited from the small size of the PHCs in SA. In contrast, the findings of previous research show that larger health care organisations, such as hospitals, are more flexible and have a higher level of readiness than PHCs or other small health care organisations [73]. Others have argued that EHRS adoption is lower in small practices than in large practices [74, 75].

### Usability

EHRS usability was also one of the facilitators to implementing a large-scale EHRS. These findings are in agreement with those by Ludwick and Doucette [70], who documented a positive relationship between the usability of the EHRS and the adoption rate. Others argued that usability issues can act as constraints to the implementation of IT in health care organisations [24, 37, 59, 76–78]. Moreover, system usability is directly associated with end-user satisfaction [34, 76]. Accordingly, EHRS end users recorded very high levels of satisfaction with system usability. Compared with previous literature, these findings are in contrast with those presented by Cresswell, Worth [79], who recorded dissatisfaction with system usability. In SA, technical issues such as EHRS usability reform are a barrier to the success of the EHRS implementation projects [80].

Feedback statements reflect the importance of involving EHRS end-users in implementation. In addition, the EHRS end users identified a few usability issues, such as the number of screens and switching between languages (Arabic and English). Other studies conducted in SA reveal that language issues are considered a barrier to EHRS implementation [80] Therefore, it is highly recommended that EHRS end users be involved and that consideration is given to their feedback and recommendations, either during software selection or when system enhancement is taking place to improve system usability. While numerous studies have documented adverse effects on the quality of care, medication errors, EHRS end-user errors, and patient safety resulting from usability issues [76, 77, 81], this study was unable to evaluate the relationship between usability issues and consequent factors such as medication errors. Therefore, further research is needed to determine this relationship in PHCs’ settings.

### Leadership and management

Strong leadership and appropriate project management play key roles in the success of large-scale projects. Our study reported that effective leadership at the senior management level can contribute to the success of the implementation of a large-scale project, and 50% of project success relies on strong and effective leadership and management. However, leadership and management issues have been documented in previous literature as barriers to EHRS implementation in SA [62]. In some cases, these issues may lead to the failure of EHRS projects [62, 82].

### Perceived usefulness

Perceived usefulness was found to be a facilitator of EHRS implementation in this study. Previous literature revealed that the EHRS was not useful and was considered a barrier to EHRS implementation [83–86]. Benefits such as data accessibility, time savings, cost reduction and improved productivity were the things that end users liked about the EHRS implemented in PHCs in SA. The findings also show that EHRS end users gave positive feedback about data accessibility, accuracy, improved productivity and time savings as a result of the system. Although these findings differ from those of several published studies [87–92] that argue that EHRS decreases staff productivity, they are consistent with those of Lorenzi, Kouroubali [12] and Cheriff, Kapur [93]. Nationally, data accessibility issues are recorded to be a barrier to EHRS implementation in SA [80].

Our findings are also consistent with those of Gagnon, Nsangou [37], Kruse, Kothman [72], Jha, DesRoches [94], and Gagnon, Desmartis [95], who found that cost reduction constitutes a major facilitator of EHRS implementation. In addition, the findings illustrated that higher perceived usefulness of an EHRS increases the end user’s willingness to use the system, which has been recorded as another facilitator of EHRS implementation. In addition to the above benefits, the EHRS contributed positively to patient outcomes. On the other hand, when examining the relationship between the perceived usefulness of the EHRS with training and support, the findings show no relationship. These findings are in disagreement with those of Carr, Zhang [96], who documented a relationship between the perceived usefulness of an EHRS with training and support.

## Conclusion

This is the first study to identify the range of facilitators to the implementation of large-scale EHRS in PHCs in Saudi Arabia. First, financial resources were found to be the most influential factor that assisted in overcoming some barriers. Second, the size of the PHC was recorded as a facilitator of successful implementation. Perceived usefulness was another identified facilitator. System usability and efficiency were documented as additional facilitators of successful EHRS implementation in Saudi PHCs. Finally, system usability and leadership and management documents are reasons to enhance the level of success of EHRS implementation.

This study provides several recommendations for decision-makers and all other EHRS implementation project teams to facilitate the implementation of a large-scale EHRS in public PHCs. First, policy-makers need to consider providing a sufficient budget for smooth implementation, particularly when decisions are being made regarding software selection. Second, centralised or semicentralised management was found to be more effective in implementing a large-scale EHRS to unify decisions, policies and procedures.

## Data Availability

The datasets generated and/or analysed during the current study are not publicly available due copyright and ownership. All primary data collected for this research belong to the researchers. The dataset includes other data that will be used for another manuscript but are available from the corresponding author on reasonable request.

## Abbreviations

MoH: Ministry of Health
EHRS: Electronic Health Record System
PHC: Primary Health Care Centres
SA: Saudi Arabia
GM: General Manager
HD: Head of Department
DHD: Deputy Head of Department
SD: Software Developer
DA: Data Analyst
FR: Financial Recourses
SPSS: Statistical Package for the Social Sciences
CISIES: The Clinical Information System Implementation Evaluation Scale
GPs: General Practitioners

## References

[1] Ortiz E, Clancy CM, Ahrq (2003). Use of information technology to improve the quality of health care in the United States. Health Serv Res.

[2] Atherton J (2011). Development of the electronic health record. AMA J Ethics.

[3] Norton PT, Rodriguez HP, Shortell SM, Lewis VA (2019). Organizational influences on healthcare system adoption and use of advanced health information technology capabilities. Am J Managed Care.

[4] Johnson KB, Neuss MJ, Detmer DE (2021). Electronic health records and clinician burnout: a story of three eras. J Am Med Inform Assoc.

[5] Biruk S, Yilma T, Andualem M, Tilahun B (2014). Health Professionals’ readiness to implement electronic medical record system at three hospitals in Ethiopia: a cross sectional study. BMC Med Inform Decis Mak.

[6] Zayyad MA, Toycan M (2018). Factors affecting sustainable adoption of e-health technology in developing countries: an exploratory survey of nigerian hospitals from the perspective of healthcare professionals. PeerJ.

[7] Ludwick Da, Doucette J (2009). Primary Care Physicians’ experience with Electronic Medical Records: barriers to implementation in a fee-for-service environment. Int J Telemedicine Appl.

[8] Deutsch E, Duftschmid G, Dorda W (2010). Critical areas of national electronic health record programs-Is our focus correct?. Int J Med Informatics.

[9] Greenhalgh T, Potts HWW, Wong G, Bark P, Swinglehurst D (2009). Tensions and Paradoxes in Electronic Patient Record Research: a systematic literature review using the Meta-narrative method. Milbank Q.

[10] Smith PD (2003). Implementing an EMR system: one clinic’s experience. Fam Pract Manag.

[11] Madore A, Rosenberg J, Muyindike WR, Bangsberg DR, Bwana MB, Martin JN (2015). Implementation of electronic medical records requires more than new software: lessons on integrating and managing health technologies from Mbarara. Uganda Healthc (Amst).

[12] Lorenzi NM, Kouroubali A, Detmer DE, Bloomrosen M (2009). How to successfully select and implement electronic health records (EHR) in small ambulatory practice settings. BMC Med Inform Decis Mak.

[13] Chao WC, Hu H, Ung CO, Cai Y (2013). Benefits and challenges of electronic health record system on stakeholders: a qualitative study of outpatient physicians. J Med Syst.

[14] Keshavjee K, Bosomworth J, Copen J, Lai J, Kucukyazici B, Lilani R, et al. Best practices in EMR implementation: a systematic review. AMIA Annu Symp Proc. 2006:982. PMC1839412 17238601

[15] Greenhalgh T, Stramer K, Bratan T, Byrne E, Mohammad Y, Russell J (2008). Introduction of shared electronic records: multi-site case study using diffusion of innovation theory. BMJ.

[16] Lorenzi NM, Smith JB, Conner SR, Campion TR (2004). The success factor Profile for clinical computer innovation. Stud Health Technol Inform.

[17] Pare G, Sicotte C, Jaana M, Girouard D (2008). Prioritizing the risk factors influencing the success of clinical information system projects. A Delphi study in Canada. Methods Inf Med.

[18] Ammenwerth E, Iller C, Mahler C (2006). IT-adoption and the interaction of task, technology and individuals: a fit framework and a case study. BMC Med Inf Decis Mak.

[19] Gagnon MP, Desmartis M, Labrecque M, Legare F, Lamothe L, Fortin JP (2010). Implementation of an electronic medical record in family practice: a case study. Inf Prim Care.

[20] Sanchez L, Savin S, Vasileva V. Key success factors in implementing electronic medical records in university hospital of Rennes. 2005.

[21] Sahay S. Special Issue on “IT and Health Care in Developing Countries”. 2001. 1–6 p.

[22] El Mahalli A (2015). Adoption and barriers to adoption of Electronic Health Records by Nurses in three Governmental Hospitals in Eastern Province, Saudi Arabia. Perspect Health Inf Manag.

[23] Alharthi H, Youssef A, Radwan S, Al-Muallim S, Zainab A-T (2014). Physician satisfaction with electronic medical records in a major saudi government hospital. J Taibah Univ Med Sci.

[24] Yusof MM, Kuljis J, Papazafeiropoulou A, Stergioulas LK (2008). An evaluation framework for Health Information Systems: human, organization and technology-fit factors (HOT-fit). Int J Med Informatics.

[25] Yusof MM, Paul RJ, Stergioulas LK, editors. Towards a framework for health information systems evaluation. System Sciences, 2006 HICSS’06 Proceedings of the 39th Annual Hawaii International Conference on; 2006: IEEE.

[26] Were MC, Meslin EM. Ethics of implementing Electronic Health Records in developing countries: points to consider. AMIA Annu Symp Proc. 2011;2011:1499–505. PMC3243215 22195214

[27] Electronic Health Records: Manual for Developing Countries [Internet]. 2007 [cited 10/12/2014]. Available from: http://www.wpro.who.int/publications/docs/EHRmanual.pdf.

[28] Simba DO, Mwangu M (2004). Application of ICT in strengthening health information systems in developing countries in the wake of globalisation. Afr Health Sci.

[29] Holroyd-Leduc JM, Lorenzetti D, Straus SE, Sykes L, Quan H (2011). The impact of the electronic medical record on structure, process, and outcomes within primary care: a systematic review of the evidence. J Am Med Inform Assoc.

[30] Barrows RC, Clayton PD (1996). Privacy, confidentiality, and electronic medical records. J Am Med Inform Assoc.

[31] Weiskopf NG, Weng C (2013). Methods and dimensions of electronic health record data quality assessment: enabling reuse for clinical research. J Am Med Inf Association: JAMIA.

[32] Ahmadian L, Nejad SS, Khajouei R (2015). Evaluation methods used on health information systems (HISs) in Iran and the effects of HISs on iranian healthcare: a systematic review. Int J Med Informatics.

[33] Sicotte C, Pare G (2010). Success in health information exchange projects: solving the implementation puzzle. Soc Sci Med.

[34] Gagnon M-P, Payne-Gagnon J, Breton E, Fortin J-P, Khoury L, Dolovich L (2016). Adoption of electronic Personal Health Records in Canada: perceptions of stakeholders. Int J Health Policy Manage.

[35] Ash JS, Fournier L, Stavri PZ, Dykstra R. Principles for a successful computerized physician order entry implementation. AMIA Annu Symp Proc. 2003:36–40. PMC1480169 14728129

[36] Xierali IM, Hsiao CJ, Puffer JC, Green LA, Rinaldo JC, Bazemore AW (2013). The rise of electronic health record adoption among family physicians. Ann Fam Med.

[37] Gagnon MP, Nsangou ER, Payne-Gagnon J, Grenier S, Sicotte C (2014). Barriers and facilitators to implementing electronic prescription: a systematic review of user groups’ perceptions. J Am Med Inform Assoc.

[38] Yontz LS, Zinn JL, Schumacher EJ (2015). Perioperative nurses’ attitudes toward the electronic health record. J Perianesth Nurs.

[39] Murray E, May C, Mair F (2010). Development and formative evaluation of the e-Health implementation toolkit (e-HIT). BMC Med Inf Decis Mak.

[40] Starfield B (1998). Primary Care: balancing health needs, services, and technology.

[41] Saad A (2004). Factors influencing the Utilisation of Public and private primary Health Care Services in Riyadh City. J King Abdulaziz Univ.

[42] Calnan M, Katsouyiannopoulos V, Ovcharov VK, Prokhorskas R, Ramic H, Williams S (1994). Major determinants of consumer satisfaction with primary care in different health systems. Fam Pract.

[43] Littlewood J, Yousuf S (2000). Primary health care in Saudi Arabia: applying global aspects of health for all, locally. J Adv Nurs.

[44] Gosden T, Forland F, Kristiansen IS, Sutton M, Leese B, Giuffrida A, et al. Capitation, salary, fee-for-service and mixed systems of payment: effects on the behaviour of primary care physicians. Cochrane Database Syst Rev. 2000(3):CD002215. 10.1002/14651858.CD002215 PMC9879313 10908531

[45] MoH MoH. The Statistics Book for the year 1432H 2011 [342]. Available from: http://www.moh.gov.sa/Ministry/Statistics/book/flash/1432/MOH_Report_1432.html.

[46] MoH MoH. The New Primary Healthcare Center Systems. 2022 [Available from: http://www.moh.gov.sa/en/Ministry/nehs/Pages/The-New-PHC-Systems.aspx.

[47] ELM. Wahed Services Form Medical Information. 2022 [Available from: https://www.elm.sa/en/e-services/Pages/wahed.aspx.

[48] Lean. Raqeem IT, Solution L. 2022 [Available from: http://www.raqeem.sa/.

[49] IQVIA. Public Health Solutions: IQVIA.com; 2022 [Available from: https://www.iqvia.com/locations/middle-east-and-africa/solutions/public-health-solutions.

[50] NTP NTP. E-health and digital tranformation. Ministry of Health; 2018. p. 124.

[51] Gugerty B, Maranda M, Rook D (2006). The clinical information system implementation evaluation scale. Stud Health Technol Inform.

[52] Lozano LA, Barthold M, Judkins-Cohn T (2014). Using the clinical information system implementation evaluation scale as a clinical implementation strategy. Comput Inf Nurs.

[53] Bryman A. Social Research Methods. OUP Oxford; 2012.

[54] Thompson SK. Sampling: Wiley; 2012.

[55] Daniel J. Sampling essentials: practical guidelines for making sampling choices. SAGE Publications; 2011.

[56] Levy PS, Lemeshow S. Sampling of populations: methods and applications. Wiley; 2013.

[57] MoH MoH. The Statistics Book for the year 1433H 2012 [342]. Available from: https://www.moh.gov.sa/Ministry/Statistics/book/Documents/1433.pdf.

[58] Ross SE, Schilling LM, Fernald DH, Davidson AJ, West DR (2010). Health information exchange in small-to-medium sized family medicine practices: motivators, barriers, and potential facilitators of adoption. Int J Med Informatics.

[59] Kruse CS, Kristof C, Jones B, Mitchell E, Martinez A (2016). Barriers to Electronic Health Record Adoption: a systematic literature review. J Med Syst.

[60] Hasanain R, Vallmuur K, Clark M. Progress and challenges in the implementation of Electronic Medical Records in Saudi Arabia: a systematic review. Health Informatics - An International Journal. 2014;3(2).

[61] Altuwaijri MM (2008). Electronic-health in Saudi Arabia. Just around the corner?. Saudi Med J.

[62] Khudair A, editor Electronic health records: Saudi physicians’ perspective. 2008 5th IET Seminar on Appropriate Healthcare Technologies for Developing Countries; 2008.

[63] Nakamura MM, Ferris TG, DesRoches CM, Jha AK (2010). Electronic health record adoption by children’s hospitals in the United States. Arch Pediatr Adolesc Med.

[64] Jotkowitz A, Oh J, Tu C, Elkin D, Pollack LA, Kerpen H (2006). The use of personal digital assistants among medical residents. Med Teach.

[65] Leon SA, Fontelo P, Green L, Ackerman M, Liu F (2007). Evidence-based medicine among internal medicine residents in a community hospital program using smart phones. BMC Med Inform Decis Mak.

[66] McAlearney AS, Schweikhart SB, Medow MA (2005). Organizational and physician perspectives about facilitating handheld computer use in clinical practice: results of a cross-site qualitative study. J Am Med Inform Assoc.

[67] Walji MF, Taylor D, Langabeer JR 2nd, Valenza JA. Factors influencing implementation and outcomes of a dental electronic patient record system. J Dent Educ. 2009;73(5):589–600. 19433534

[68] Koivunen M, Hatonen H, Valimaki M (2008). Barriers and facilitators influencing the implementation of an interactive internet-portal application for patient education in psychiatric hospitals. Patient Educ Couns.

[69] Jha AK, Bates DW, Jenter C, Orav EJ, Zheng J, Cleary P (2009). Electronic health records: use, barriers and satisfaction among physicians who care for black and hispanic patients. J Eval Clin Pract.

[70] Ludwick DA, Doucette J (2009). Adopting electronic medical records in primary care: lessons learned from health information systems implementation experience in seven countries. Int J Med Inform.

[71] Ash JS, Bates DW (2005). Factors and forces affecting EHR system adoption: report of a 2004 ACMI discussion. J Am Med Inform Assoc.

[72] Kruse CS, Kothman K, Anerobi K, Abanaka L (2016). Adoption factors of the Electronic Health record: a systematic review. JMIR Med Inform.

[73] Jones EB, Furukawa MF (2014). Adoption and use of electronic health records among federally qualified health centers grew substantially during 2010-12. Health Aff (Millwood).

[74] DesRoches CM, Worzala C, Joshi MS, Kralovec PD, Jha AK (2012). Small, nonteaching, and rural hospitals continue to be slow in adopting electronic health record systems. Health Aff (Millwood).

[75] Ancker JS, Singh MP, Thomas R, Edwards A, Snyder A, Kashyap A (2013). Predictors of success for electronic health record implementation in small physician practices. Appl Clin Inform.

[76] Khajouei R, Wierenga PC, Hasman A, Jaspers MW (2011). Clinicians satisfaction with CPOE ease of use and effect on clinicians’ workflow, efficiency and medication safety. Int J Med Informatics.

[77] Middleton B, Bloomrosen M, Dente MA, Hashmat B, Koppel R, Overhage JM (2013). Enhancing patient safety and quality of care by improving the usability of electronic health record systems: recommendations from AMIA. J Am Med Inform Assoc.

[78] Hettinger AZ, Melnick ER, Ratwani RM (2021). Advancing electronic health record vendor usability maturity: Progress and next steps. J Am Med Inform Assoc.

[79] Cresswell KM, Worth A, Sheikh A (2012). Comparative case study investigating sociotechnical processes of change in the context of a national electronic health record implementation. Health Inf J.

[80] AlSadrah SA (2020). Electronic medical records and health care promotion in Saudi Arabia. Saudi Med J.

[81] Zahabi M, David BK, Manida S (2015). Usability and safety in Electronic Medical Records Interface Design:a review of recent literature and Guideline Formulation. Hum Factors.

[82] Boonstra A, Broekhuis M (2010). Barriers to the acceptance of electronic medical records by physicians from systematic review to taxonomy and interventions. BMC Health Serv Res.

[83] Nguyen L, Bellucci E, Nguyen LT (2014). Electronic health records implementation: an evaluation of information system impact and contingency factors. Int J Med Informatics.

[84] Boonstra A, Versluis A, Vos JF (2014). Implementing electronic health records in hospitals: a systematic literature review. BMC Health Serv Res.

[85] Clarke MA, Belden JL, Koopman RJ, Steege LM, Moore JL, Canfield SM (2013). Information needs and information-seeking behaviour analysis of primary care physicians and nurses: a literature review. Health Info Libr J.

[86] Tubaishat A (2018). Perceived usefulness and perceived ease of use of electronic health records among nurses: application of Technology Acceptance Model. Inform Health Soc Care.

[87] Zandieh SO, Yoon-Flannery K, Kuperman GJ, Hyman D, Kaushal R. Correlates of expected satisfaction with electronic health records in office practices by practitioners. AMIA Annu Symp Proc. 2008:1190. 18998904

[88] Handel DA, Hackman JL (2010). Implementing electronic health records in the emergency department. J Emerg Med.

[89] Miller RH, West C, Brown TM, Sim I, Ganchoff C (2005). The value of electronic health records in solo or small group practices. Health Aff (Millwood).

[90] Chen C, Garrido T, Chock D, Okawa G, Liang L (2009). The Kaiser Permanente Electronic Health Record: transforming and streamlining Modalities of Care. Health Aff.

[91] Huerta TR, Thompson MA, Ford EW, Ford WF (2013). Electronic health record implementation and hospitals’ total factor productivity. Decis Support Syst.

[92] Redd TK, Read-Brown S, Choi D, Yackel TR, Tu DC, Chiang MF (2014). Electronic health record impact on productivity and efficiency in an academic pediatric ophthalmology practice. J Am Association Pediatr Ophthalmol Strabismus.

[93] Cheriff AD, Kapur AG, Qiu M, Cole CL (2010). Physician productivity and the ambulatory EHR in a large academic multi-specialty physician group. Int J Med Informatics.

[94] Jha AK, DesRoches CM, Campbell EG, Donelan K, Rao SR, Ferris TG (2009). Use of Electronic Health Records in U.S. hospitals. N Engl J Med.

[95] Gagnon MP, Desmartis M, Labrecque M, Car J, Pagliari C, Pluye P (2012). Systematic review of factors influencing the adoption of information and communication technologies by healthcare professionals. J Med Syst.

[96] Carr AS, Zhang M, Klopping I, Min H (2010). RFID Technology: implications for Healthcare Organizations. Am J Bus.

